# Two-Dimensional Self-Consistent Radio Frequency Plasma Simulations Relevant to the Gaseous Electronics Conference RF Reference Cell

**DOI:** 10.6028/jres.100.036

**Published:** 1995

**Authors:** Dimitris P. Lymberopoulos, Demetre J. Economou

**Affiliations:** Plasma Processing Laboratory, Department of Chemical Engineering, University of Houston, Houston, TX 77204-4792

**Keywords:** argon discharge, plasma modeling, plasma processing, reference cell, rf discharges, two-dimensional simulation

## Abstract

Over the past few years multidimensional self-consistent plasma simulations including complex chemistry have been developed which are promising tools for furthering our understanding of reactive gas plasmas and for reactor design and optimization. These simulations must be benchmarked against experimental data obtained in well-characterized systems such as the Gaseous Electronics Conference (GEC) reference cell. Two-dimensional simulations relevant to the GEC Cell are reviewed in this paper with emphasis on fluid simulations. Important features observed experimentally, such as off-axis maxima in the charge density and hot spots of metastable species density near the electrode edges in capacitively-coupled GEC cells, have been captured by these simulations.

## 1. Introduction

Low pressure (0.13 Pa to 1333 Pa), cold (gas temperature 300 K to 500 K), weakly ionized (degree of ionization 10^−6^ to 10^−1^) glow discharge plasmas are used extensively in the processing of electronic materials, especially for etching and deposition of thin films [1]. They also find application in surface modification (e.g., hardening, corrosion resistance) and lighting. In reactive gas plasmas, electrons decompose the flowing feedstock gas into radicals and ions. In plasma deposition, radicals adsorb on the wafer surface where they react to deposit a thin film. The film microstructure and properties (e.g., density, resistivity) can be influenced by energetic ion bombardment which occurs naturally on all surfaces exposed to the plasma. In plasma etching, radicals adsorb and react on the wafer to form volatile products which desorb and are pumped away by a vacuum system. The surface chemistry can be strongly modified by energetic ion bombardment. Ions bombard the wafer preferentially along the vertical direction, enhancing the reaction rate and inducing anisotropy which is critical for delineating sub-half micron patterns in advanced microelectronic device manufacturing. Controlling the flux, energy distribution and angular distribution of ions and neutrals bombarding the wafer is of paramount importance in plasma systems. Also, the uniformity of these fluxes over large diameter (> 200 mm) wafers is critical for the success of industrial plasma processing equipment.

Unfortunately, glow discharge plasmas are extremely complex systems in which a plethora of interdependent parameters can influence the process, often in a subtle way. Thus, slight variations in reactor design and operating conditions can result in large variations in system performance. The Gaseous Electronics Conference (GEC) Reference Cell was conceived to serve as a common platform for experimental and modeling studies in different laboratories [2]. The Reference Cell is thought to be a well-characterized system in which fundamental studies of plasma behavior can be conducted. Experimental data obtained in the Cell are also useful for benchmarking plasma simulations, which in turn provide insight into the plasma dynamics. This synergistic experimental-modeling approach is crucial for furthering our understanding of plasma systems and for the development of predictive simulation tools which are useful for the design and optimization of new reactors.

The Reference Cell can be operated in two configurations: capacitively- or inductively-coupled plasma. Most studies related to the Cell so far are for the capacitively-coupled configuration, a schematic of which is shown in Fig. 1a. The bottom electrode is usually powered with a 13.56 MHz power supply, and is separated from the grounded walls by thin insulators. This system has azimuthal symmetry, but the area of the powered electrode is much smaller than that of the grounded electrode. Since the time-average sheath voltage in capacitively-coupled systems scales with the inverse ratio of the electrode areas [1], a large voltage drop appears in the sheath over the powered electrode with a much smaller voltage across the sheath over the grounded electrode. Sometimes, a push-pull rf drive configuration is used in which both the bottom and the top electrodes are powered with a rf voltage of the same amplitude but 180° out of phase. This results in a discharge that is symmetric not only azimuthally but also axially. The time-average sheath voltages over the top and bottom electrodes will then be identical in magnitude. The capacitively coupled Cell, henceforth to be designated as the Gaseous Electronics Conference Capacitively Coupled Plasma (GEC-CCP) Cell, is usually operated as a relatively high pressure (6.67 Pa to 133 Pa) low density (charge density < 10^17^ m^−3^) plasma (LDP).

Recently, the Cell has been operated with an inductive coil to generate a low-pressure (< 6.67 Pa) high charge density (> 10^17^ m^−3^) plasma (Fig. 1b). High density plasma (HDP) sources are becoming increasingly important in microelectronics as device dimensions continue to shrink. Low pressure provides more directional ion bombardment and better plasma uniformity over large diameter wafers. High plasma density ensures that the etch or deposition rate are comparable to those found in high pressure LDP systems. The inductively coupled Cell will henceforth be designated as GEC-ICP.

Most experimental studies with the GEC cell have used noble gas plasmas (Ar, He), because they are simpler than reactive gases (Cl_2_, SF_6_). This also facilitates comparison of experimental data with simulation results since the electron impact collision cross sections are known and the noble gas plasma reactions are better characterized as compared to reactive gases. Furthermore, noble gas plasmas are electropositive (i.e., the negative ion to electron density ratio is much less than unity, (*n*_–_/*n_e_*<<1) and as such are simpler to experiment with, both in the laboratory and on the computer as compared to electronegative (*n*_–_/*n_e_*>1) plasmas.

## 2. Problem Statement

Given the reactor configuration shown in Fig. 1a or Fig. 1b and a set of operating parameters (feedstock gas composition and flow rate, gas pressure, voltage or current waveform applied to the electrode), determine the following: The space and time variation of electron, ion, and neutral species densities and velocities, the flux and energy distribution of ions and neutrals bombarding the electrodes and their uniformity across the electrodes, the power deposited into the plasma, and the potential and current distribution in the system. If the reactor is loaded with a wafer to etch, one is in addition interested in the etch rate, uniformity, anisotropy (shape of microscopic features etched into the wafer), selectivity, and radiation damage. The level of detail one can obtain depends on the type of simulation used. For example, fluid simulations can’t provide the species distribution functions but only averages over the distribution.

## 3. Plasma Simulation

Modeling and simulation of glow discharge plasmas has emerged as a tool for enhancing one’s intuition about the physicochemical processes occurring in the plasma, for understanding the complex spatiotemporal plasma dynamics, and for assisting in the design of new reactors or the optimization of existing ones. Two-dimensional plasma reactor simulations have been reported in the literature in recent years [3–5]. However, these works focused on the transport and reaction of neutrals only (neutral transport and reaction models). The electron density was *assumed* to have a uniform or Bessel function profile, and the electron temperature was not calculated as a function of space and time in the reactor. These studies did not solve the problem of neutral radical transport and reaction in a self-consistent manner. The radical source terms (by electron-impact dissociation, for example) were estimated and the conservation equations for mass, momentum, and energy transport of the neutrals were solved to obtain the fluid velocity profiles, neutral gas temperature and the concentration distribution of radicals. Charged particle transport was not considered, and the effect of plasma gas composition (different from the feedstock gas composition) on the plasma properties was not accounted for.

Up until a few years ago, simulations that solved for the rf plasma dynamics (using the so-called glow discharge models) were confined to one spatial dimension (1-D) [6–19]. In addition, most of these simulations did not solve for the transport and reaction of neutrals. Self-consistent rf plasma simulations which solve for the coupled effects of charged and neutral species transport have only been reported within the past few years in 1-D [16,18,19] and 2-D [20–22]. Two dimensional simulations are particularly useful since they can address the important issue of plasma uniformity and the spatiotemporal plasma dynamics along both the radial and axial direction. As of this writing, most 2-D simulations do not include neutral transport and chemistry and have considered noble gases (argon and helium) only, not reactive gas plasmas [23–27]. We are aware of only a few 2-D plasma simulations which couple the neutral transport and chemistry with the glow discharge in a self-consistent manner [20–22]. Several more groups are now working on this problem. In view of the above discussion, multidimensional self-consistent plasma reactor simulation is still at an early stage of development.

There are three kinds of glow discharge simulations: fluid, kinetic and hybrid. Fluid simulations integrate moments of the Boltzmann equation (see below) describing species density, momentum and energy conservation. They require some assumptions regarding the species distribution function to achieve closure of the equations. Kinetic simulations, such as Particle-In-Cell with Monte Carlo Collisions (PIC-MCC) [28–30] or Direct Simulation Monte Carlo (DSMC) [31] follow the motion of a number of superparticles accounting for their collisions using Monte Carlo techniques. Kinetic simulations yield the particle distribution functions as an output of the simulation. They are considered more accurate than fluid simulations in the sense that they can be used at low pressures (mean free path comparable to or longer than the characteristic length scale, or Knudsen number *Kn* = *λ*/*L* >1) or for highly nonequilibrium situations. However, there is some evidence that fluid simulations can perform well even at low pressures for which they should be considered suspect [32]. Kinetic simulations are computationally intensive as compared to fluid simulations. Hybrid simulations have been developed [16, 22] in an attempt to preserve the accuracy of kinetic simulations and at the same time reduce the computational burden.

## 4. Model Formulation

This paper emphasizes the fluid simulation approach since all studies of the GEC Cell are based fully or largely on this approach. The reader is referred to the literature for examples of kinetic plasma simulations using PIC/MCC [28–30] or DSMC [31].

### 4.1 The Fluid Equations

In general, species (electrons, ions, neutrals) transport can be described by the Boltzmann equation

$$\frac{\partial f}{\partial t}+u\cdot \nabla_{r}f+h\cdot \nabla_{u}f=\frac{\delta f}{\delta t},$$ (1)

which is a continuity equation in phase space (***r***, ***u***), where ***r*** is the spatial location vector and ***u*** is the species velocity vector. The acceleration is ***h*** = ***F***/*m*, where ***F*** is the force acting on the species and *m* is the mass. For a particle with charge *q*, ***F*** = *q* (***E*** + ***u***×***B***), is the Lorentz force, where ***E*** and ***B*** are the electric field and magnetic induction, respectively. The right hand side of Eq. (1) is the so-called collision integral, which accounts for the effect of collisions on the velocity distribution function (VDF) *f*. The VDF is defined such that the number of particles d*N* with velocities between ***u*** and ***u* +** d***u*** in a given volume d***r*** of configuration space is given by

dN=fdrdu. (2)

Consequently, the number density as a function of position can be obtained as

n(r)≡∫fdu, (3)

where the integral is over the velocity space.

The right hand side of Eq. (1) is an integral containing *f* which is the unknown [33]. Eq. (1) is extremely difficult to solve because it is an integrodifferential equation in three space dimensions (*x*, *y*, *z*), three velocity dimensions (*u_x_, u_y_, u_z_*) and time (*t*). Kinetic simulations such as DSMC and PIC/MCC solve for the species distribution function by integrating the Boltzmann equation using pseudoparticles. On the other hand, fluid simulations solve equations for “average” quantities such as the directed velocity. The fluid equations are obtained from the Boltzmann Eq. (1) by first multiplying that equation by *ϕ*, where *ϕ* is a constant or a function of the velocity ***u***, and then integrating over the velocity space to obtain the “average” of *ϕ*. The resulting equations are called moments of the Boltzmann equation [34].

When *ϕ* = 1, the species *i* number density continuity equation (where *i* can be electrons *i = e*, positive ions *i* = +, negative ions *i* = −, or neutrals *i = n*) is obtained as the zeroth moment,

$$\frac{\partial n_{i}}{\partial t}+\nabla \cdot (n_{i}v_{i})=\sum_{j}R_{ij}.$$ (4)

where *n_i_* and ***v****_i_* are the density and directed velocity of species *i*, respectively. *R_ij_* is the rate of production or loss of species *i* due to the volumetric reaction *j*.

The first moment is the (vector) momentum equation which can be derived by setting *ϕ·*= *m****u***,

$$\frac{\partial }{\partial t}(n_{i}m_{i}\nu_{i})+\nabla \cdot (n_{i}m_{i}v_{i}v_{i})=-\nabla P_{i}+n_{i}m_{i}h_{i}-n_{i}m_{i}v_{i}v_{mi},$$ (5)

where *v_mi_* is an *effective* momentum exchange frequency of species *i* and *P_i_* is the partial pressure given by *P_i_* = *n_i_****κ****T_i_*, where ***κ*** is the Boltzmann constant and *T_i_* is the species temperature. Equation (5) assumes that the pressure tensor is isotropic which appears to be a good approximation in the absence of strong magnetic fields. In Eq. (5), the terms represent (in order from left to right) local acceleration, convective acceleration (inertia), motion due to pressure gradient, motion due to the presence of force fields and momentum exchange due to collisions with the background species.

When the terms on the left hand side of Eq. (5) are negligible (see Ref. [17] for a discussion of this point), one obtains

$$v_{i}=\frac{1}{m_{i}v_{mi}}\left(q_{i}E-\frac{1}{n_{i}}\nabla \;(n_{i}\;\kappa T_{i})\right).$$ (6)

Since the flux for each carrier is ***J****_i_* = *n_i_****v****i* one can write

$$J_{i}=q_{i}\mu_{i}n_{i}\;E-D_{i}\nabla n_{i},$$ (7)

where *µ_i_* and *D_i_* are the particle’s mobility and diffusivity respectively, and the species temperature has been taken outside the differential as if the temperature were constant. Equation (7) is known as the drift-diffusion approximation and is often used in place of the full momentum Eq. (5) for simplicity [6–10]. The drift-diffusion approximation has been questioned at low pressures and in the sheath where species inertia may not be negligible [34]. Comparing Eqs. (6) and (7) one obtains

$$\mu_{i}=\frac{q_{i}}{m_{i}v_{mi}}$$ (8)

$$D_{i}=\frac{\kappa T_{i}}{m_{i}v_{mi}}.$$ (9)

Combination of Eqs. (8) and (9) results in Einstein’s relation,

$$\frac{D_{i}}{\mu_{i}}=\frac{\kappa T_{i}}{q_{i}}.$$ (10)

Although electrons are mobile enough to respond to the variations of the electric field at 13.56 MHz (i.e., *v_me_*≫*ω*, and the electron inertia can be neglected), ions are massive and can’t follow the field faithfully. Recognizing this fact, Richards et al. [8] introduced an equation for an “effective” field to which the ions respond.

$$\frac{\partial E_{i}^{\text{eff}}}{\partial t}=v_{mi}(E-E_{i}^{\text{eff}}).$$ (11)

That way, the drift-diffusion Eq. (7) can be used for both electrons and ions, except that the actual electric field is replaced by the effective field, in the case of ions. Of course, when the full ion momentum Eq. (5) is used, Eqs. (6) and (7) become immaterial.

The second moment of the Boltzmann equation is the (scalar) energy conservation equation which can be derived by setting ϕ=mu⋅u/2=ℐ, where ℐ is the total (kinetic plus thermal) energy. For particle *i*, ${ℐ}_{i}=m_{i}v_{i}^{2}/2+U_{i}$ with *U_i_* the thermal energy

$$\frac{\partial n_{i}{ℐ}_{i}}{\partial t}+\nabla \cdot (n_{i}v_{i}{ℐ}_{i})=-\nabla \cdot P_{i}v_{i}+n_{i}m_{i}h_{i}\cdot v_{i}+\nabla \cdot K_{i}\nabla T_{i}-\sum_{j}R_{ij}H_{ij}.$$ (12)

Here *K_i_* is the thermal conductivity and *H_ij_* is the energy loss due to collision of type *j*. In order from left to right, the terms in Eq. (12) represent the time rate of change of total energy, convective transport of energy, rate of work done by pressure forces, energy exchange with the force field, energy transport by conduction, and energy loss due to collisions.

An equation for the thermal energy *U_i_* (For a Maxwellian distribution *U_i_* = 3/2***κ****T_i_*) can be obtained by taking the dot product of ***v****_i_* with the momentum Eq. (5), and subtracting the resulting equation from the total energy Eq. (12) [34]. However, a simplification is frequently made in plasma simulations, namely that $U_{i}\gg m_{i}v_{i}^{2}/2$, and ${ℐ}_{i}$ in Eq. (12) is simply replaced by *U_i_*. The resulting equation, written here for electrons (*i* = *e*), reads,

$$\frac{\partial }{\partial t}\left(\frac{3}{2}n_{e}\kappa T_{e}\right)+\nabla \cdot q_{e}+eJ_{e}\cdot E+3v_{me}\frac{m_{e}}{M}n_{e}\kappa (T_{e}-T_{g})+\sum_{j}R_{ej}H_{ej}=0$$ (13)

with the total electron energy flux given by

$$q_{e}=-K_{e}\nabla T_{e}+\frac{5}{2}\kappa T_{e}J_{e}.$$ (14)

The thermal conductivity of a monatomic “gas” is given by

$$K_{i}=\frac{3}{2}\kappa D_{i}n_{i}.$$ (15)

In Eq. (13), the electron energy loss term has been decomposed into losses due to elastic collisions, and inelastic collisions (last two terms on left hand side). Some authors write Eq. (13) in terms of the thermal energy *U_i_*, i.e., they don’t make the substitution *U_i_* = 3/2***κ****T_i_* [27, 35]. This way, they have an equation in terms of the mean electron energy instead of one in terms of electron temperature. The two formalisms are equivalent.

Depending on the approximations made, different sets of the equations shown above are used by different authors. Most often, the drift diffusion approximation is made for both electrons and ions [6–10, 20]. Other authors solve the full momentum equations for either electrons or ions (using drift-diffusion for the other species) [25,26,36] or for both electrons and ions [11].

### 4.2 Electron-Impact Rate Coefficients and Transport Properties

The above derivation of the fluid equations tacitly assumes that the velocity distribution function is Maxwellian (introducing a “temperature” automatically implies a Maxwellian distribution). Although the VDF of ions and neutrals can be non-Maxwellian at low pressures, it is the electron energy distribution function (EEDF) which is of primary concern. Experimental measurements in capacitively coupled rf plasmas have shown non-Maxwellian EEDFs [37]. It is thought that the most important effect of a non-Maxwellian distribution would be on the electron-impact reaction rate coefficients, especially those for high threshold energy processes (e.g., excitation, ionization) which are sensitive to the shape of the tail of the EEDF, or processes with sharp resonances.

Rate coefficients for electron impact reactions are needed as input to the fluid equations. For example, when applied to electrons in an argon discharge, the right hand side of Eq. (4) reads,

$$\sum_{j}R_{ej}=k_{i}Nn_{e}+k_{si}n_{\mathrm{Ar}*}n_{e}+k_{mp}n_{\mathrm{Ar}*}^{2},$$ (16)

where *N*, *n_e_* and *n*_Ar*_ are ground state argon, electron, and metastable species density, respectively. The relevant reactions producing electrons and the corresponding rate coefficients are shown in Table 1 (reactions 2, 3, and 6). Electrons are produced by direct ionization of ground state Ar, by ionization of metastable atoms (two-step ionization) and by metastable-metastable collisions. The electron-impact rate coefficients are calculated by an expression of the form,

$$k_{j}=\int_{0}^{\infty }\sigma_{j}(\varepsilon )u_{e}(\varepsilon )f(\varepsilon )d\varepsilon ,$$ (17)

where *k_j_* is the rate coefficient for process *j*, **σ***_j_* is the corresponding collision cross section, *f*(***ε***) is the EEDF, and ***ε*** is the electron energy $\varepsilon =m_{e}u_{e}^{2}/2$ *u_e_* being the magnitude of the electron velocity, *u_e_* = |***u****_e_*|. In addition, electron transport properties (momentum exchange collision frequency, mobility, diffusivity, etc.) are needed. These can also be obtained as the appropriate integrals over the distribution function [38].

Kinetic simulations can predict the EEDF and Eq. (17) can be integrated to calculate the rate coefficients. In fluid simulations, the electron-impact rate coefficients are expressed as a function of the mean electron energy. Two approaches are used: (a) the EEDF is assumed, for example Maxwellian or Druyresteyn distribution and, knowing the cross sections, Eq. (17) is integrated directly to find *k_j_* as a function of average energy, or (b) A spatially-independent (0-D) Boltzmann equation solver is used [either finite difference or finite element or Monte Carlo solution of Eq. (1)] to calculate the distribution function (for a given gas composition) and in turn the electron-impact reaction rate coefficients and transport properties as a function of *E/N*. At the same time the mean electron energy is calculated as a function of *E/N*. The results are combined to eliminate *E/N* and express the rate coefficients and the transport properties as a function of mean electron energy (or temperature; in the case of non-Maxwellian distribution, the “temperature” is defined as 2/3 of the average energy, $T_{e}=2\bar{\varepsilon }/3\kappa$). Since the latter is one of the dependent variables that is obtained as part of the solution [(see Eq. (13))], this representation is very convenient. It should be noted that this approach tacitly assumes that the EEDF in the rf discharge of interest is similar to that which would be obtained in a DC swarm experiment. This approach apparently works fairly well [39]. A more complicated approach in which the time dependence of the EEDF is also taken into account has also been proposed [16]. The charged species mobility is usually assumed constant, although it can be expressed as a function of the mean electron energy. Finally, the charged species diffusivity is commonly obtained from the mobility and the species temperature using the Einstein relation, Eq. (10).

In the hybrid approach of Kushner and coworkers [18, 22], the electron energy fluid equation [Eq. (13)] is dropped. Instead, the time-average electron-impact rate coefficients and transport properties are obtained by a 2-D Monte Carlo simulation. Therefore, no assumptions need be made regarding the distribution function.

Some 1-D RF [40, 41] and 2-D RF [24] and DC [42] glow discharge simulations applied the so-called local field approximation. In this approximation, the electron energy Eq. (13) is dropped. The electron impact rate coefficients and transport properties are expressed as a function of *E*/*N*. It is assumed that these quantities at a given point in the discharge and at a given time during the rf cycle are equal to the DC values that would be obtained using the same *E*/*N* as existed at that point in the discharge and that particular time in the rf cycle. This approximation does not allow for nonlocal behavior of the EVDF and is particularly bad at low pressure, for beam electrons (emitted by ion bombardment of the electrodes), and for describing electron transport in the sheath.

Note that including Eq. (13) allows for nonlocal electron transport to be captured since the rate coefficients are a function of mean electron energy and not *E*/*N*. And it is well known that fluid models predict the electron energy to peak at the plasma-sheath interface, whereas the electric field peaks right at the electrode surface. Comparisons of fluid with kinetic simulations [33] show that fluid models underpredict the nonlocal electron behavior. For example fluid simulations predict a sharper electron energy peak compared to kinetic simulations.

In addition to electron transport and reaction coefficients, one also needs rate coefficients for ion-neutral and neutral-neutral reactions and the transport properties (mobility, diffusivity, etc.) of the heavy (ions, neutrals) species. In fluid models, the ion energy distribution function is usually assumed to be Maxwellian with a temperature equal to the gas temperature. When the full momentum equation (Eq. 5) for ions is solved, a drifting Maxwellian distribution is assumed. An ion energy (Eq. 12) or a corresponding temperature equation have not been incorporated in the fluid models so far, but work towards that goal is in progress [43].

### 4.3 Maxwell’s Equations

Self-consistent plasma simulations must solve for the electromagnetic fields which develop in the reactor. Maxwell’s equations relate the electromagnetic field intensity and flux density vectors to each other and to the sources of the field, the electric charges and currents. In differential form, Maxwell’s equations are [44]:

$$\nabla \times E=-\frac{\partial B}{\partial t},$$ (18)

$$\nabla \times H=J+\frac{\partial D}{\partial t},$$ (19)

∇⋅D=ρ, (20)

∇⋅B=0. (21)

In the above equations ***E***, ***D***, ***H***, ***B***, ***J***, and ***ρ*** are the electric field intensity, electric field flux, magnetic field intensity, magnetic field flux, current density, and charge density, respectively. These equations are not independent. For example Eq. (20) can be obtained by taking the divergence of Eq. (19) and making use of the equation of continuity of electrical charge, ∇ · ***J*** + ∂*ρ*/∂*t* = 0. The above equations are augmented with the constitutive relations ***B*** = *µ****H*** and ***D*** = *ε****E*** where *µ* is the permeability and ε is the permittivity of the medium.

In the absence of any time varying magnetic fields (as is the case of typical GEC-CCP cells), ∂***B***/∂*t* = 0 and Faraday’s law Eq. (18) suggests that the electric field can be written as the divergence of a scalar. Thus,

E=−∇V, (22)

where *V* is the electric potential. Substituting Eq. (22) into Eq. (20) and assuming a dielectric constant independent of position, yields an equation relating the Laplacian of the potential to the charge density, referred to as Poisson’s equation,

$$\nabla^{2}V=-\rho /\varepsilon .$$ (23)

When solving the Poisson equation over a domain that includes different material properties, the dielectric constant should be kept inside the differential.

The equation for the propagation of electromagnetic fields is obtained from the Maxwell equations as

$$\nabla^{2}E-\mu \varepsilon \frac{\partial^{2}E}{\partial t^{2}}-\mu \sigma \frac{\partial E}{\partial t}=0,$$ (24)

where use has also been made of Ohm’s law, ***J*** = **σ*E*** (***σ*** is the conductivity of the medium). Equation (24) is the three-dimensional wave equation for an absorbing medium. Similar equations can be derived for the other field quantities, ***D***, ***B***, and ***H*** [44]. Equation (24) can be solved for the fields as a boundary value problem. Alternatively, the magnetic vector potential ***A*** formulation may be used. Based on the general notion that the divergence of the curl of a vector is zero (∇·∇×***v*** = 0) one may write the magnetic field of Eq. (21) as the *curl* of a vector ***A***,

B=∇×A. (25)

Using Eq. (25), Faraday’s law Eq. (18) can be written as

$$\nabla \times \left(E+\frac{\partial A}{\partial t}\right)=0.$$ (26)

Then by using the identity ∇×∇*V* = 0, where *V* is the electric potential one obtains

$$E+\frac{\partial A}{\partial t}=-\nabla V,$$ (27)

where the negative sign is introduced on the right-hand side so that *V* becomes the electric potential in a static situation, when ***A*** is independent of time.

One can now derive an equation for ***A***,

$$\nabla^{2}A-\mu \varepsilon \frac{\partial^{2}A}{\partial t^{2}}=-\mu J.$$ (28)

where ***J*** is the current density giving rise to the electromagnetic fields. For azimuthally symmetric systems for which the applied current has an azimuthal component only, *A_r_* and *A_z_* are zero, and only the *A_ϑ_* component of the magnetic vector potential survives. In this case one also has ∂*V/*∂ϑ = 0 and, using Eq. (27), the azimuthal component of the electric field is given by

$$E_{\vartheta }=-\frac{\partial A_{\vartheta }}{\partial t},$$ (29)

and *E_r_* and *E_z_* are given by the respective radial and axial gradients of the electric potential *V*.

It is quite common to replace the time-harmonic electromagnetic quantities *A*_ϑ_, *E*_ϑ_ and *J*_ϑ_ with their phasor representation $A_{\vartheta },=\tilde{A}e^{j\omega t},E_{\vartheta }=\tilde{E}e^{j\omega t}$ and $J_{\vartheta }=\tilde{J}e^{j\omega t}$ respectively, where $\tilde{A}$, $\tilde{E}$ and $\tilde{J}$ are the corresponding (complex) amplitudes which depend only on space, and *ω* is the applied frequency. Equation (29) then becomes

$$\tilde{E}=-j\omega \tilde{A}$$ (30)

Recognizing that *A*_ϑ_ is only a function of (*r*, *z*) Eq. (28) is simplified to

$$\frac{1}{r}\frac{\partial }{\partial r}\left(r\frac{\partial \tilde{A}}{\partial r}\right)+\frac{\partial^{2}\tilde{A}}{\partial z^{2}}+(\omega^{2}\mu \varepsilon -r^{-2})\tilde{A}=-\mu \tilde{J},$$ (31)

where the complex current density is given by $\tilde{J}=\tilde{\sigma }\tilde{E}$ and $\tilde{\sigma }$ is the complex conductivity of the medium. Making the cold plasma approximation, the complex plasma conductivity ***σ***_p_ is given by

$$\sigma_{p}=\frac{n_{e}q^{2}}{m_{e}(V_{\text{eff}}+j\omega )}$$ (32)

where *m*_e_ and *v*_eff_ are the electron mass and *effective* momentum-transfer collision frequency, respectively. The plasma dielectric constant *ε*_p_ is obtained from

$$\varepsilon_{p}=\varepsilon_{0}-j\frac{\sigma_{p}}{\omega },$$ (33)

where *ε*_0_ is the permittivity of vacuum.

Equation (31) can be solved in the whole domain (including plasma, reactor walls, etc.) by a finite difference [22, 45] or finite element method [46]. Once $\tilde{A}$ has been determined the azimuthal electric field is retrieved by using the following equation

Eϑ=Re(−jωA˜ejωt). (34)

The power deposition $\bar{W}$ is equal to

W¯(r,z)=12{Re(σ˜E˜2)}. (35)

The inductive power deposition given by Eq. (35) should be added to the right hand side of Eq. (13) in the GEC/ICP reactor case. The assumption of Ohm’s law implies that the power deposition is by collisional processes. At low operating pressures (< 1.33 Pa) noncollisional power deposition can take place [47, 48]. However, the same formulation as for the collisional case may be used, except that an effective electron collision frequency must be used in Eq. (32) [49].

### 4.4 Boundary and Initial Conditions

Boundary conditions are difficult to specify in fluid simulations, partly because the physics of the problem is not well understood (in which case even kinetic simulations have a problem) and partly because the resulting expressions may not be convenient to use for solving the fluid equations. Boundary conditions are needed for the species density, velocity, and temperature (or energy). In addition, boundary conditions for solving Maxwell’s equations are necessary.

The boundary condition on electron density takes the form of essentially a “mass balance” at the wall. The electron flux at the wall equals the thermal flux (assuming that electrons striking the wall are fully absorbed) minus the secondary electron emission flux. The latter equals the positive ion flux striking the wall ***J***_+_ times the secondary electron emission coefficient γ_+_. The thermal flux of electrons is given by *ν*_t_*n*_e_/4 where ν*_t_* is the thermal velocity, equal to (8***κ****T*_e_/π*m*_e_)^1/2^, and *n*_e_ is the electron density, both evaluated at the wall. Therefore,

$$J_{e}=\frac{1}{4}\sqrt{\frac{8\kappa T_{e}}{\pi m_{e}}}n_{e}\hat{n}-\gamma_{+}J_{+}.$$ (36)

Combining Eq. (36) with the expression for the electron flux ***J***_e_ as given by Eq. (7), one obtains a rather complex expression in terms of electron density and temperature, ion density and the species mobility at the wall. Some authors have used the simpler condition *n*_e_ = 0. However, both kinetic and fluid simulations have shown that a substantial electron density can exist near the electrode, especially during the anodic part of the rf cycle [33]. Eq. (36) is written here for one type of ion striking the wall. For a mutli-ion plasma more terms accounting for the different ions should be included on the right hand side of Eq. (36).

For positive ions, the flux at the electrode is dominantly due to drift because of the large value of the electric field,

$$J_{+}=\mu_{+}n_{+}E^{\text{eff}},$$ (37)

where ***E***^eff^ is given by Eq. (11). Setting *n*_+_ = 0 at the electrode is not appropriate, neither it is desirable from the numerical point of view. Although ions are presumably completely neutralized at the wall (Auger neutralization for example), this happens only within a distance ~10^−10^ m from the wall, which would require resolution of a steep ion boundary layer [6]. Negative ions are not energetic enough to overcome the potential barrier at the wall, hence *n*_−_ = 0. For neutrals, a flux boundary condition is used. For example for Cl atoms recombining on the wall with probability γ_Cl→Cl2_, one has

$$J_{\mathrm{Cl}}=\frac{\gamma_{\mathrm{Cl}\to {\mathrm{Cl}}_{2}}}{4}\sqrt{\frac{8\kappa T_{g}}{\pi m_{\mathrm{Cl}}}}n_{\mathrm{Cl}}\hat{n}-\gamma_{{\mathrm{Cl}}^{+}\to \mathrm{Cl}}J_{{\mathrm{Cl}}^{+}},$$ (38)

where $\gamma_{{\mathrm{Cl}}^{+}\to \mathrm{Cl}}$ is the probability of ion neutralization on the wall, usually assumed unity. This expression states that the net flux of Cl at the surface equals the flux of Cl atoms recombining (or in general reacting) on the surface minus the flux of Cl atoms forming on the surface by neutralization of Cl^+^ ions. The latter term is not important in LDP, but can become very important in HDP sources. For highly reactive walls, the concentration at the surface may be set equal to zero. For example, if metastable atoms are assumed to deactivate with unity probability upon striking the wall, n_*_ = 0. Chantry [50] has proposed an extrapolation method to specify the boundary condition at low pressures.

The boundary condition for electron mean energy is written essentially in the form of an energy balance at the electrode [6,19,23].

$$q_{e}=\left(\frac{5}{2}\kappa T_{e}\right)\frac{1}{4}\sqrt{\frac{8\kappa T_{e}}{\pi m_{e}}}n_{e}\hat{n}-\gamma_{+}\left(\frac{5}{2}\kappa T_{\mathrm{se}}\right)J_{+}.$$ (39)

When γ_+_ is zero, combination of Eqs. (36) and (39) with the expression for ***q***_e_ (Eq. (14)) suggests that the electron temperature gradient is zero at the electrode. Some workers have assumed a constant electron temperature at the electrode (e.g., *κT*_e_ = 1 eV). This is convenient from the numerical point of view, but the actual value of the electron temperature is unknown. In addition, the temperature at the wall may be a function of time. Kinetic simulations do not have the problem of specifying a temperature boundary condition; indeed the electron energy as a function of time at the wall is an output of the simulation [33]. The boundary condition on the electric potential is specified as *V* = 0 on grounded walls. On the rf driven electrode the potential is

$$V=V_{\mathrm{DC}}+V_{\mathrm{RF}}\;\sin\;\omega t.$$ (40)

The self-bias voltage *V*_DC_ has to be found as part of the solution. The usual approach is to specify V_rf_ and then adjust the value of *V*_DC_ during the simulation so that the net charged particle (electrons and ions) current at the capacitively coupled electrode is zero [27, 35]. Gogolides and Sawin [17] and Dalvie et al. [23] have used a current boundary condition on the driven electrode instead of Eq. (40), assuming singly charged ions,

$$eJ_{+}-eJ_{e}+\varepsilon_{0}\frac{\partial E}{\partial t}=I_{0}\sin\;\omega t.$$ (41)

Equation (41) implies that the total current, composed of the particle (ion and electron) and the displacement currents, is forced to be *I*_0_ sin *ωt*. On insulators the following boundary condition is specified [21, 23],

$$eJ_{+}-eJ_{e}+\varepsilon_{0}\frac{\partial E}{\partial t}=\pm \frac{\varepsilon_{i}}{d_{i}}\frac{\partial V_{i}}{\partial t}$$ (42)

which assumes no surface conduction on the insulator. Here *V*_i_, *d*_i_, and *ε*_i_ are the voltage drop across the insulator, thickness and dielectric constant of the insulator, respectively. Eq. (42) is a current continuity equation analogous to Eq. (41) and implies that the total current (conduction plus displacement current from the plasma) equals the displacement current through the insulator. Boundary conditions used in fluid simulations have been discussed by Wilcoxson and Manousiouthakis [51, 52].

Different sets of initial conditions have been used with a varied degree of success. For example, a uniform charge density many orders of magnitude lower than the expected steady-state value may be specified at *t* = 0. Sometimes parabolic-like charged species distributions seem to work better. In any case it is important to satisfy the Poisson equation at *t* = 0; a potential of *V* = 0 everywhere is a common choice. The electron energy can be specified as spatially uniform initially. Convergence is not guaranteed; however, a converged solution can be used as an initial condition for another simulation at a different set of nearby operating conditions. A formal continuation scheme can also be applied to conduct parametric investigations [53]. Of course, the final periodic steady-state solution should be independent of the choice of initial conditions, unless physically acceptable multiple steady states exist.

## 5. Computational Aspects

Glow discharge simulations are computationally intensive because they are “stiff” in both space and time. Spatial stiffness results from the fact that rapid changes in the dependent variables occur near and inside the sheath, which forms naturally over all surfaces in contact with the discharge. Under high-pressure low-density conditions the sheath thickness may be 10–100 times less than the characteristic discharge dimension, depending on pressure, applied voltage, excitation frequency, etc. The situation is particularly acute in HDP reactors which have a sheath thickness of 100–1000 times less than the discharge dimension. This is because high plasma density results in a small (10s of µm) Debye length *λ*D, and the sheath thickness is only 10s of *λ*D. Another problem results from the fact that the sheaths are “highly convective” (or “advective”) in nature, meaning that the “drift” current under the influence of the electric field [first term on the right hand side of Eq. (7)] far surpasses the diffusion current [second term on the right hand side of Eq. (7)]. Numerical handling of highly convective flows in an accurate and efficient manner is still an area of active research in computational fluid dynamics (CFD) [34]. Traditionally, upwind differencing has been used in finite difference discretizations or the Streamlined Upwind Petrov-Galerkin method in finite element approximations (SUPG-FEM) [54]. For glow discharge simulations the Scharfetter-Gummel exponential scheme, first used in modeling of solid state semiconductor devices, has been used extensively [55]. Unfortunately, artificial diffusion is inevitably introduced by these methods. Flux Corrected Transport (FCT) methods [56, 57] are designed to minimize artificial diffusion, but specification of the antidiffusive fluxes poses a problem. The Donor Cell Method (DCM) [22] and the More Accurate FCT (MAFCT) [26] have also been used for plasma simulation.

Stiffness in time is a problem since electron, ion, and neutral species response times are <1 ns, 1 µs to 10 µs, and 10 ms to 100 ms or longer, respectively. The smallest operational time scale that needs to be resolved is that of the applied excitation frequency, which is typically 13.56 MHz (a period of 73.4 ns). Assuming that the time step has to be at least 20 times smaller than the excitation frequency and that the simulation has to continue for 100 ms to capture neutral chemistry, one would require some 3×10^7^ time steps to complete the simulation. Clearly this is a tremendous computational task. To make matters even worse, the time step must usually be much smaller than that required to resolve the excitation frequency. For example to assure stability, explicit time integration schemes [58] must have a time step which is limited by the Courant-Friedrich-Lewy (CFL) condition [36], Δ*t*<Δ*x/ν*_max_, where Δ*t* is the time step size, Δ*x* is the grid spacing and *ν*_max_ is the maximum species velocity. Explicit integration is straightforward to execute but may require many time steps (>1,000) per cycle to reach the periodic steady state. To relax the CFL constraint implicit or semi-implicit integration schemes [58] of the transport equations (Eqs. (4), (5), and (12)) are usually employed [11, 19, 22, 59]. Implicit integration requires fewer time steps, but results in a system of complex nonlinear equations that may be solved by iterative methods.

For high charge densities, the dielectric relaxation (DR) time [22,34,36] imposes severe limitations on time step (even more so than the CFL condition) when the Poisson Eq. (23) is integrated explicitly

$$\Delta t_{\mathrm{DR}}=\varepsilon_{0}/(e\mu_{e}n_{e}+e\mu_{+}n_{+}).$$ (43)

For HDP simulations, for example, where the charge density is in the range of 10^17^ m^−3^ to 10^18^ m^−3^, one is constrained to Δ*t*_DR_<10^−12^ s! Implicit or semi-implicit schemes can overcome this limitation. When Poisson’s equation, Eq. (23), is solved separately from the charge continuity equations (either implicitly or explicitly) the time advancement of the potential is explicit in nature as the charge density is evaluated at time *t*. Therefore, this approach is bound to advance in time with at most Δ*t*_DR_. However, Ventzek et al. [22] devised a semi-implicit update technique for the electric potential that allowed the time steps to exceed Δ*t*_DR_ by 1–2 orders of magnitude. The method is based on approximating the charge density at a future time, *ρ*(*t* + Δ*t*), by relying on the present values of *ρ*(*t*) and its derivative (d*ρ*/d*t*)*_t_* (i.e., *ρ*(*t* +Δ*t*) ≈ *ρ*(*t*) + d*ρ* /d*t*)*_t_*Δ*t*). Stewart et al. [59] overcame the dielectric relaxation time step limitation by removing the strong coupling between the electron continuity and Poisson’s equation. This was achieved by substituting the Laplacian of the electric potential (i.e., ∇^2^*V*) appearing in the electron continuity equation (Eq. (4) written for electrons) by the right hand side of Eq. (23).

Formal acceleration schemes based on the Newton-Raphson method [17, 19, 53] or heuristics based on extrapolation [22, 60–62] have been used to accelerate convergence to the periodic steady state. It has been estimated that these techniques can speed up convergence by many orders of magnitude.

**Fig. 2:**
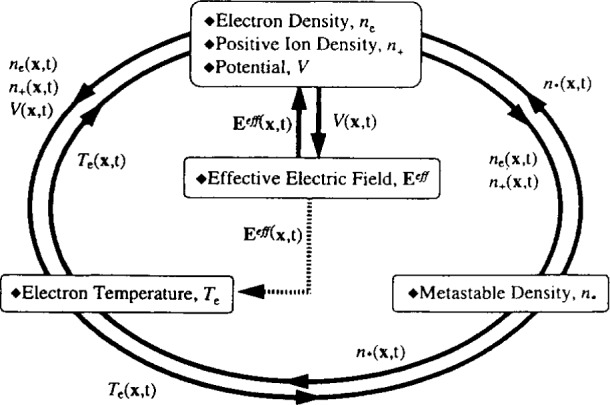
Modular approach used to perform a 2-D plasma simulation of an argon rf discharge.

In order to decouple the disparate time scales of electron and neutral transport, Kushner and co-workers [22, 62] and Lymberopoulos and Economou [19, 20] used a modular approach which can be thought of as an equation splitting technique. An example, used for a capacitively-coupled argon discharge, is shown in Fig. 2. The glow discharge module includes the electron and ion density continuity [Eq. (4)] and the drift-diffusion [Eq. (7)] equations which are solved simultaneously with the Poisson equation [Eq. (23)]. The electron energy (temperature) equation [Eq. (13)] is then updated using the new charge densities and fields. In turn, the neutral metastable density equation [Eq. (4)] is solved on a staggered mesh which does not have to be as fine as the grid used for the glow discharge and electron temperature modules. The simulation then returns to the glow discharge module with updated values of the electron energy and metastable density. The equations for the effective electric field to which the ions respond [Eq. (11)] are also solved periodically. Information is thus cycled back-and-forth among the modules until convergence. This approach can be extended to the GEC-ICP cell by including a module that solves for the azimuthal electric field [Eq. (31)]. This has been done for simulating the GEC cell [62] and other HDP reactors [46].

## 6. Results and Discussion

### 6.1 GEC/CCP Cell

Although there have been quite a few 1-D simulations of the GEC-CCP cell (see paper by Govindan and Meyyappan in this Special Issue), the authors are aware of only three published 2-D simulations of the GEC/CCP cell [20, 21, 27], and one published simulation [62] of the GEC-ICP cell. Preliminary results of a hybrid 2-D simulation of the GEC-CCP cell were reported by Pak and Riley [63]. Other works in two-dimensional geometries that are relevant to the GEC-CCP cell are the papers of Dalvie et al. [23], and Wu and co-workers [26]. Still more 2-D capacitively-coupled rf glow discharge simulations have been reported by Tsai and Wu [24], Paschier and Goedheer [35], and Wilcoxson and Manousiouthakis [52], all using the fluid approximation, and by Vahedi et al. [30] who used a PIC-MCC approach.

**Fig. 3:**
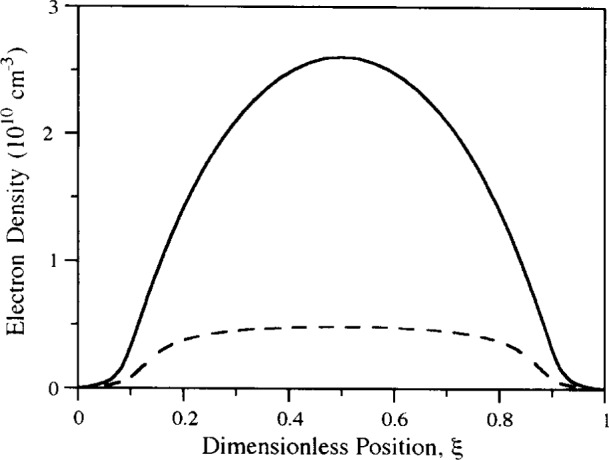
Electron density between the electrodes of a capacitively coupled rf argon discharge. Solid line: including metastables in the calculation in a self-consistent manner. Dashed line: without metastables. Conditions: 133.3 Pa, 300 K, 100 V peak-to-peak, 13.56 MHz rf voltage (from Ref. [19]).

Lymberopoulos and Economou [20, 21] simulated an argon discharge including the transport of neutral metastables in a self-consistent manner. The problem was simplified by considering only one (lumped) metastable state, and the relatively simple chemistry shown in Table 1. They used a 0-D Monte Carlo simulation to express the electron-impact reaction rate coefficients as a function of mean electron energy [19]. One of the results of that work was the significance of metastable neutral atoms in sustaining the discharge by a two-step ionization process (i.e., excitation to the metastable level first followed by ionization of that level, see Reactions 1 and 3 in Table 1). The importance of two-step ionization was recognized earlier in connection with dc discharges (positive column) [64, 65]. Figure 3 shows a comparison of the time-average electron density obtained by a 1-D rf (13. 56 MHz) simulation of an argon discharge with (solid line) and without (dashed line) metastables [19]. Including metastables results in several times higher electron density which is then in accord with experimental measurements [66]. Metastables have an ionization threshold of only 4.14 eV as compared to 15.7 eV for direct ionization from the ground state. Although the metastable density is orders of magnitude smaller than the ground state species, the corresponding ionization coefficient is orders of magnitude greater; in fact it is such that the two-step ionization is the dominant mechanism for electron production under these conditions (133.3 Pa pressure). The importance of metastables in sustaining the discharge points to the possible effects that minute amounts of impurities (e.g., air or moisture from a vacuum leak) can have, since these impurities can quench metastables very effectively.

**Fig. 4:**
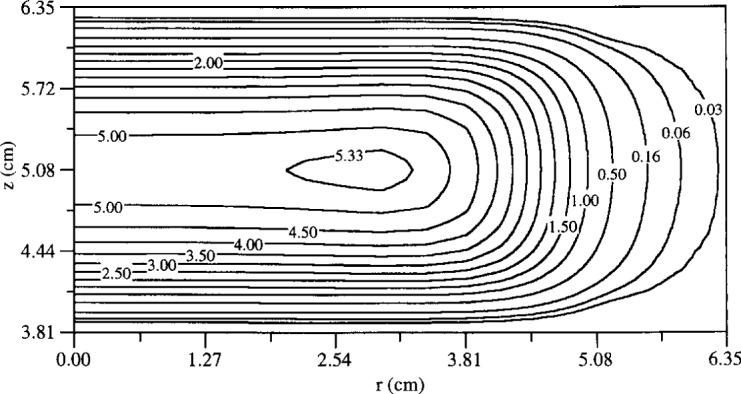
Electron density (in 10^15^ m^−3^) in an argon discharge in the GEC Cell of Fig. 1a for a symmetric (push-pull) rf drive. Conditions: 133.3 Pa, 300 K, 60 V peak-to-peak, 13.56 MHz rf voltage (from Ref. [20]).

The time-average electron density distribution from a 2-D rf (13.56 MHz) simulation of an argon discharge at a pressure of 133.3 Pa is shown in Fig. 4 [20]. A push-pull scheme was used to power the Cell which resulted in a symmetric discharge (no dc bias). Only part of the Cell of Fig. 1a is shown for clarity. Near the reactor centerline the electron density has a cosine-like profile similar to that of the 1-D simulation of Fig. 3. A peak in electron density is observed in the radial direction. The electron density drops off drastically beyond the electrode edge, i.e., the plasma is well confined. This is because of the relatively high pressure (133.3 Pa) low voltage operation (60 V peak-to-peak) and the fact that the discharge is symmetric.

**Fig. 5:**
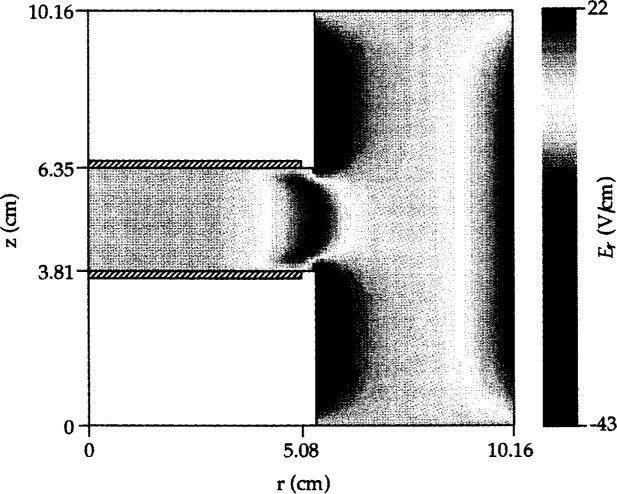
Radial electric field distribution in the GEC Cell of Fig. 1a for symmetric (push-pull) rf drive. Conditions: 133.3 Pa, 300 K, 60 V peak-to-peak, 13.56 MHz rf voltage.

The radial peak in charge density has been attributed to the synergistic effect between the axial and radial electric fields near the edge of the electrodes. A radial (space charge) electric field develops as the more mobile electrons escape the plasma between the two electrodes into the surrounding chamber. The radial electric field extends over a region ~1 cm inwards (towards the centerline) from the electrode edges (Fig. 5). Away from the edge the electric field is directed axially and heats the electrons primarily near the plasma-sheath interface. Near the edge, the electrons acquire extra energy due to the radial electric field and hence enhance the ionization resulting in a radial peak in the charge density. Radial peaks in electron and ion density have been measured in the GEC-CCP Cell by Overzet and Hopkins [67].

**Fig. 6:**
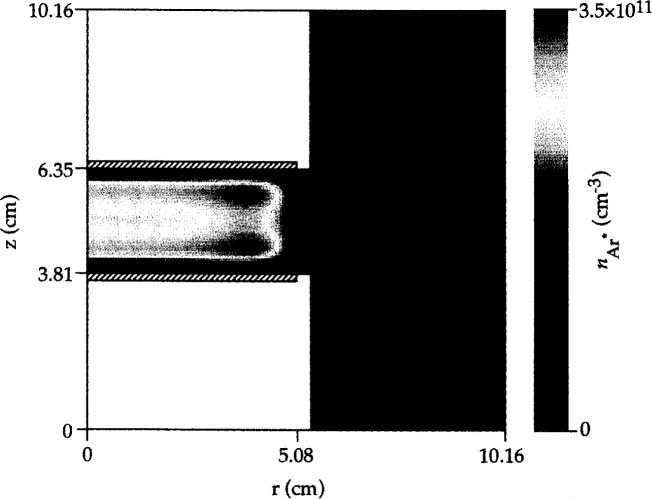
Argon metastable density in the GEC Cell of Fig. 1a for symmetric (push-pull) rf drive. Conditions: 133.3 Pa, 300 K, 60 V peak-to-peak, 13.56 MHz rf voltage.

**Fig. 7:**
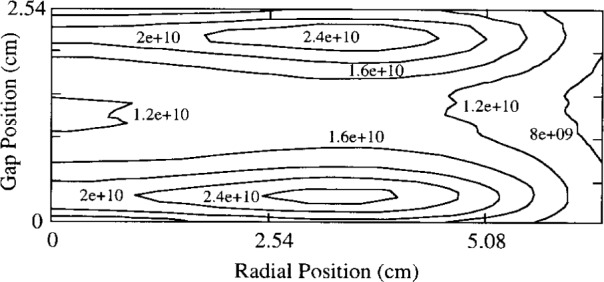
Measured helium metastable (2 ^1^Σ) density in the GEC Cell. Conditions: 133.3 Pa, 300 V peak-to-peak, 13.56 MHz rf voltage (from Ref. [66]).

The enhanced electron energy near the electrode edges is also reflected in the neutral metastable density profiles: hot spots in metastable density develop near the edges as seen in Fig. 6. The metastable density is depressed along the midplane between the electrodes because the main loss mechanism for metastables is quenching by electrons (reaction 5 in Table 1); and the electron density peaks along the midplane. A structure similar to that shown in Fig. 6 has been observed experimentally by Greenberg and Hebner [66] in the Cell for a corresponding helium metastable state (see Fig. 7).

**Fig. 8:**
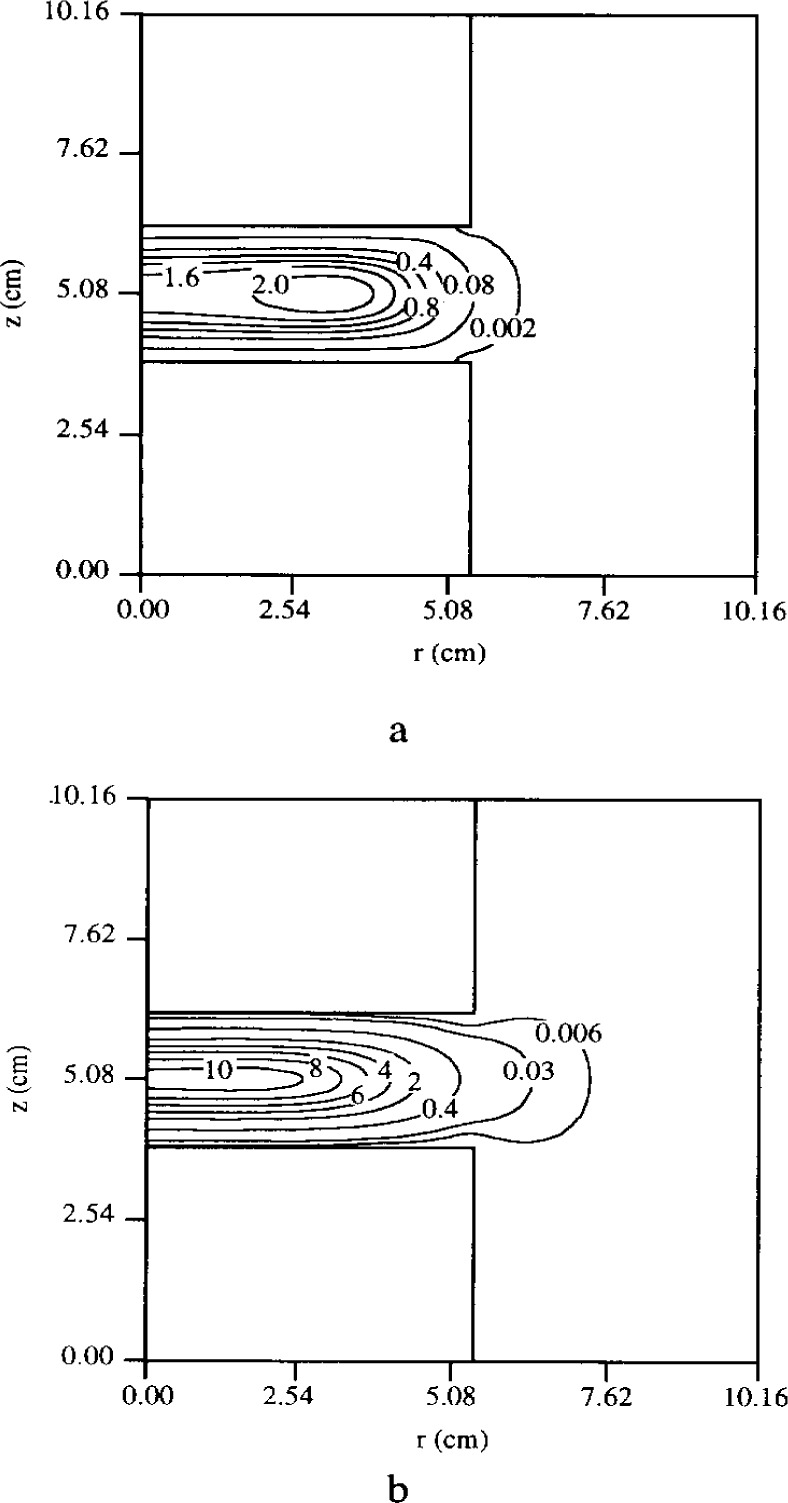
(a) Rate of ionization of argon by the direct mechanism (reaction 2 of Table 1). Contour values should be multiplied by 10^19^ m^−3^ s^−1^. (b) Rate of ionization of argon by the two-step mechanism (reaction 3 of Table 1). Contour values should be multiplied by 10^18^ m^−3^ s^−1^. Conditions: 13.3 Pa, 300 K, 60 V peak-to-peak, 13.56 MHz rf voltage.

The importance of metastables in contributing to ionization at a pressure of 133.3 Pa was discussed in connection with Fig. 3. Figure 8 shows the electron production rate due to direct ionization (Reaction 2 in Table 1) and two-step ionization (reaction 3 in Table 1), for a pressure of 13.3 Pa. Two-step ionization is only 10 % of the direct ionization in this case. As the pressure is lowered, the mean electron energy increases. Higher electron energies favor more endothermic reactions (direct vs two-step ionization). Even at this lower pressure metastables make a significant contribution to ionization. Ventzek et al. [62] have shown that the two-step ionization continues to be significant even down to 1.33 Pa. Apparently even at 1.33 Pa in the GEC-ICP discharge [62], the electron energy is not high enough for direct ionization to completely dominate. Another reason is that the metastable density *n*_Ar*_ depends relatively weakly on pressure; it appears to change by only a few times as the pressure changes by a factor of 100 (compare results of Ref. [19], [21], and [62] for pressures of 133.3 Pa, 13.3 Pa, and 1.33 Pa respectively). Thus, the ratio of metastable to ground state density increases with decreasing pressure and that makes the two-step ionization contribution significant.

**Fig. 9:**
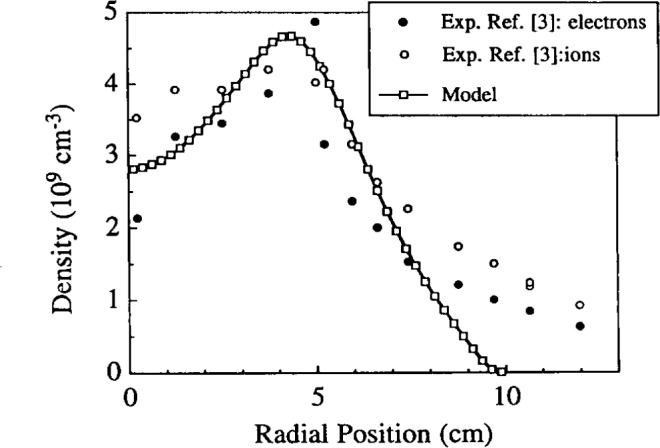
Comparison of experimental data of electron (solid dots) and ion (open circles) density [(L. J. Overzet and M. B. Hopkins, Appl. Phys. Lett. 63, 2484 (1993)] with model predictions (line with open squares), in an rf argon discharge in the GEC Cell. Conditions: 33.3 Pa, 200 V peak-to-peak, 13.56 MHz rf voltage (from Ref. [27]).

Boeuf and Pitchford [27] used an approach very similar to that of Lymberopoulos and Economou [20, 21] except that Boeuf and Pitchford did not include metastable neutral transport. Their method of solution was based on a finite difference approximation of the spatial derivatives using the Scharfetter-Gummel exponential scheme. They used a uniform 41×41 grid. For an electrode spacing of 2.54 cm, this corresponds to a grid spacing of 0.6 mm between the electrodes. An explicit time integration was used to advance the simulation to the periodic steady-state reached after some 1000 rf cycles (13.56 MHz) with 500 time steps per cycle. They compared their simulation results with the data of Overzet and Hopkins [67] and they found good agreement with those measurements (Fig. 9). The agreement was much better at 33.3 Pa (shown in Fig. 9) than at 13.3 Pa which is surprising since Boeuf and Pitchford did not include metastables in their calculation and the metastable contribution is expected to be more significant at the higher pressure.

**Fig. 10:**
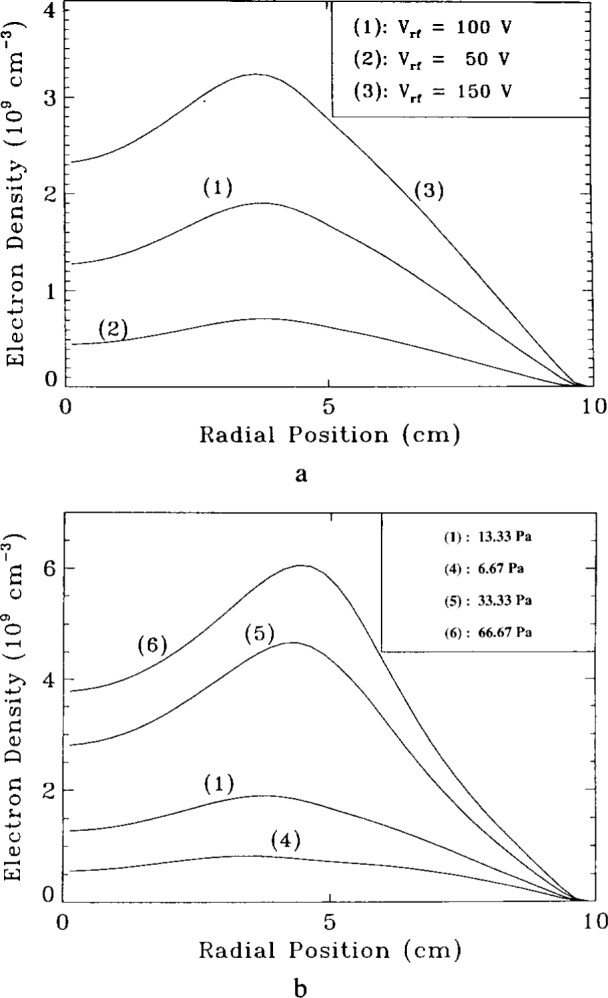
Time-average radial profiles of electron density in an agron discharge in the GEC Cell for different values of rf peak voltage (a) and pressure (b). The profiles are for the midplane between the electrodes. Pressure in (a) was 13.3 Pa. 13.56 MHz rf peak voltage in (b) was 100 V (from Ref. [27]).

The time-average radial electron density profiles at a distance of 1.25 cm from the powered electrode are shown in Fig. 10a as a function of applied rf voltage amplitude, and in Fig. 10b as a function of pressure [27]. It is clear that higher voltages and/or pressures enhance the off-axis radial peak in electron density. As voltage increases the radial electric field increases as well which in turn increases the ionization rate near the radial edge of the plasma. Also, as the pressure is lowered diffusion becomes more facile smoothing the concentration gradients.

**Fig. 11:**
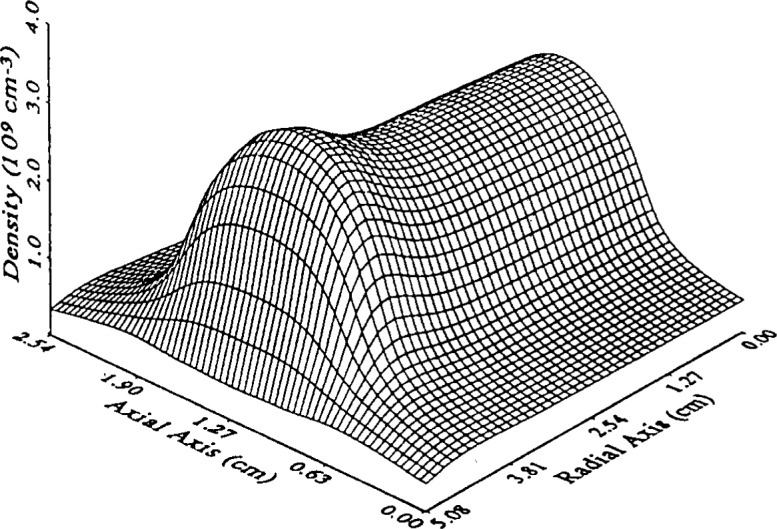
Time-average ion density in a helium 13.56 MHz rf discharge at 133.3 Pa and 150 V cm^−1^ (vacuum) field between the electrodes.

Young and Wu [26] simulated a 13.56 MHz helium discharge in a geometry relevant to the GEC-CCP cell. They truncated the surrounding chamber by placing a cylindrical solid wall confining the discharge to a radius of 5.08 cm (2 in). The electrode spacing in their simulation was 2.54 cm (1 in) as in the Reference Cell. They used a fluid approximation with the full momentum and energy equations (Eqs. (5) and (12)) for the electrons and the drift-diffusion approximation with an effective electric field (Eqs. (7) and (11)) for ions. They did not consider metastable transport in their simulation. Electron-impact reaction rate coefficients and transport properties were obtained by a 0-D DC Monte Carlo simulation. Figure 11 shows that a peak in the radial profile of the time-average ion density appears in this case as well. Young and Wu attributed this peak to the presence of a radial electric field which provides extra heating of the electrons in the region close to the radial wall. In this case the radial electric field is present because of the sheath which forms naturally over the radial wall. It is interesting to note that their result (i.e., off-axis radial peaks in charge density) is similar to that obtained in the open GEC-CCP geometry of Fig. 1 where the radial confining wall is away from the electrodes [20,21,27].

**Fig. 12:**
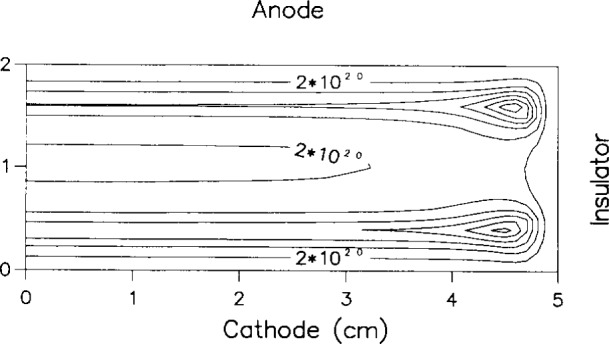
Time-average ionization rate in an argon discharge at 66.7 Pa and 0.175 A, 13.56 MHz rf peak current (see Eq. (41)). Contour values increase in steps of 2×10^20^ m^−3^ s^−1^ (from Ref. [23]).

**Fig. 13:**
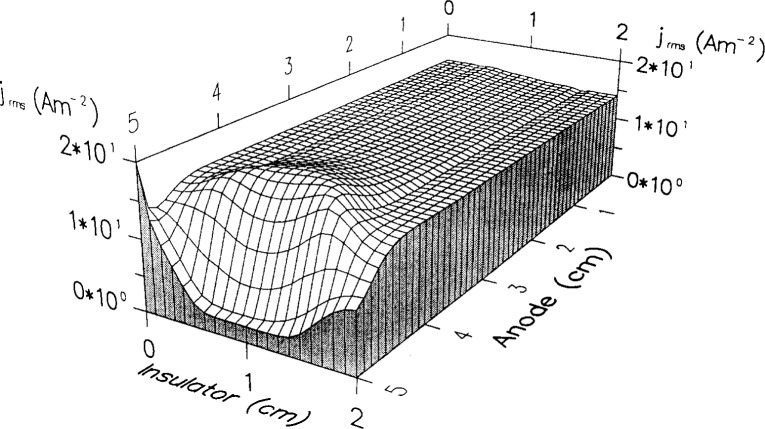
Root-mean-square current density in an argon discharge at 66.7 Pa and 0.175 A, 13.56 MHz rf peak current (see Eq. (41)) (from Ref. [23]).

Dalvie et al. [23], also observed an off-axis radial peak in the charge density in a 2-D simulation of a reactor with a radial confining wall. They used the fluid equations with the drift-diffusion approximation (no effective field for ions), and Arrhenius rate expressions for the ionization and excitation rates of argon as a function of electron temperature. The authors did not consider metastable transport. They used a sinusoidal total current boundary condition at the powered electrode (Eq. (41) with *I*_0_ = 0.175 A, and *ω* = 2 *πv*, *v* = 13.56 MHz). The radial wall was an insulator (quartz) which was grounded on the opposite side of that exposed to the plasma. When the insulator was “thick,” the discharge was nearly symmetric since the resistance was too high for appreciable (displacement) current to flow through the insulator. The time-average ionization rate at a pressure of 66.7 Pa is shown in Fig. 12. Local maxima in ionization are seen near the discharge corners which the authors attributed to extra electron heating at the corners due to the focusing of the rf current by the radial sheath. The focusing of the current is seen in Fig. 13 which displays the root-mean-square current density in the cell. As in the case of the simulations of Young and Wu [26] and Lymberopoulos and Economou [20, 21], the presence of a radial sheath contributes to extra electron heating. Since the spatial distribution of ionization and excitation rates in argon are similar (both are high threshold processes), the local maxima in Fig. 12 would produce a local enhancement in the density of metastable species, should the authors have included metastables in their calculation. This would be consistent with Fig. 6.

### 6.2 GEC/ICP Cell

Kushner and coworkers [62] have developed a comprehensive 2-D simulation of the GEC-ICP cell shown in Fig. 1(b). They used a modular approach which is an extension of their previous work on 1-D glow discharge simulations [18] and which is in the same spirit of the modular approach (Fig. 2) followed by Lymberopoulos and Economou [20]. Fluid equations were used to compute the electron, ion and neutral species densities. An electromagnetics module solved for the azimuthal electric field distribution [Eq. (31)] and Eq. (18) was used to obtain the magnetic fields. Poisson’s equation was solved implicitly simultaneously with the charged species density continuity equations. The electromagnetic fields were used in a 2-D Monte Carlo simulation performed in regular intervals during the progress of the overall simulation to determine the time-average electron transport properties and rate coefficients of electron-impact reactions. Also, a Navier-Stokes hydrodynamics module was used to calculate the gas velocity distribution. Information was cycled among the modules until convergence. This is a hybrid simulation in the sense that the electron impact rate coefficients are calculated by a kinetic Monte Carlo scheme and not by a (fluid) energy equation.

**Fig. 14:**
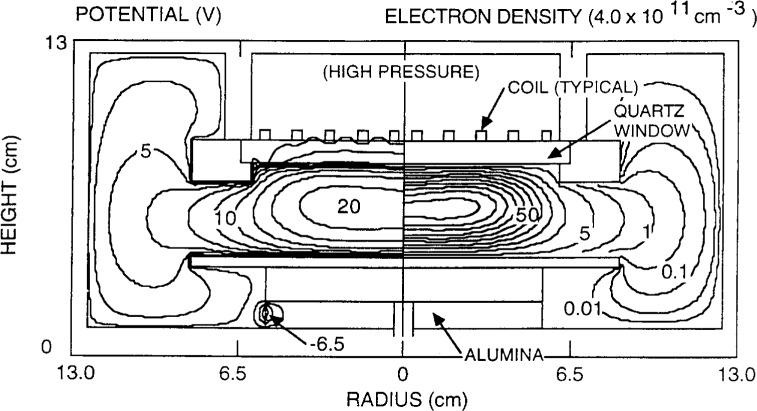
Time-average electron density (right) and potential (left). 1.33 Pa inductively-coupled argon plasma in the GEC Reference Cell of Fig. 1b (from Ref. [62]). A contour value of 100 corresponds to 4×10^17^ m^−3^ electron density.

**Fig. 15:**
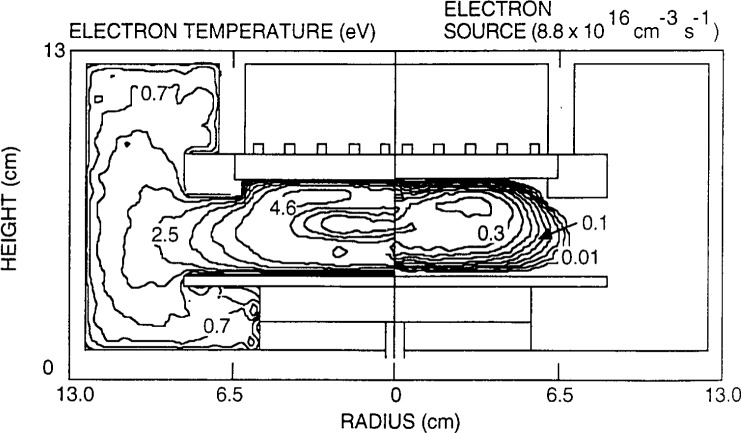
Time-average rate of ionization (right) and electron temperature (left). 1.33 Pa inductively-coupled argon plasma in the GEC Reference Cell of Fig. 1b (from Ref. [62]).

The results of the GEC-ICP simulation by Ventzek et al. [62] are shown in Figs. 14 and 15. Figure 14 shows the time-average electron density (right) and plasma potential (left). The stove-top coil dimensions are also shown in Fig. 14. The plasma is rather well confined despite the low operating pressure of 1. 33 Pa. This is consistent with experimental measurements in the GEC-ICP cell. The electron density drops by an order of magnitude a small distance beyond the edge of the coil, and decays to very small values in the surrounding chamber. The plasma potential peaks at around 20 V. The negatively charged alumina support at the lower part of the reactor is also shown. The electrons are fairly hot under the coils (Fig. 14, left) but they cool off drastically in the surrounding chamber. The ionization is confined in the main plasma volume under the coils (Fig. 15) since both the electron density and temperature drop off sharply as a function of radius beyond the coil edge. Good agreement was obtained for the electron density as a function of power between the computed and measured values [68].

**Fig. 16:**
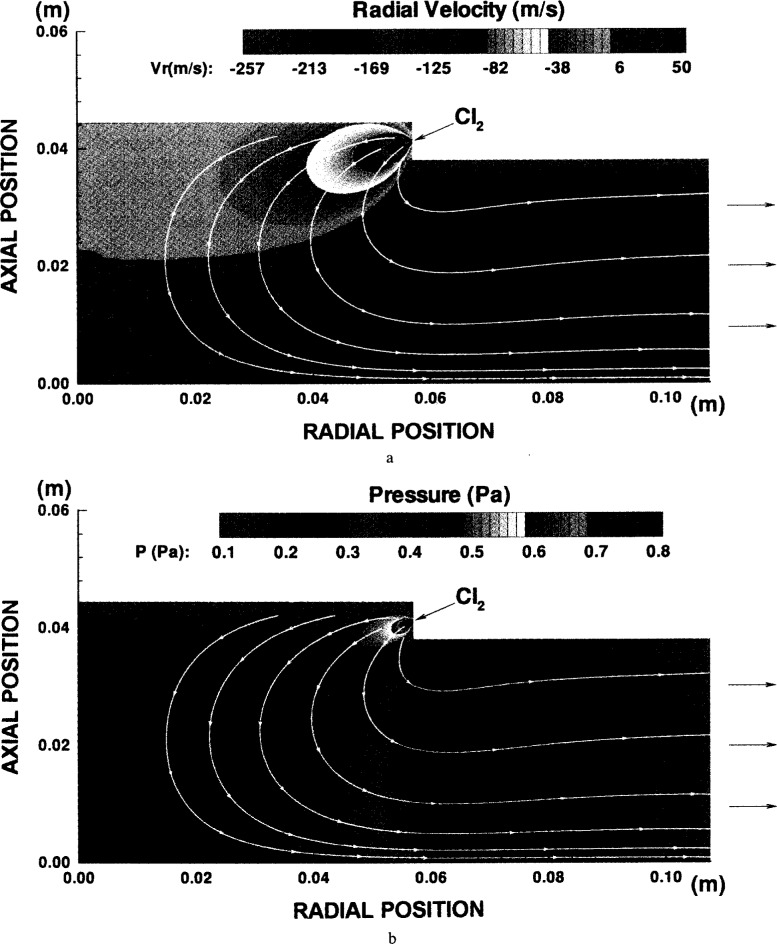
Radial velocity component (a) and pressure distribution (b) in the GEC Cell of Fig. 1b. Flow rate of pure Cl_2_ 140 sccm. The (0, 0) point represents the center of the surface of the lower electrode.

Economou and Bartel [69] have used a kinetic Direct Simulation Monte Carlo (DSMC) method to simulate gas flow and pressure distribution in the GEC-ICP cell. DSMC is expected to be more accurate than a fluid simulation at pressures down to 0. 13 Pa at which the mean free path of species exceeds the reactor spacing (*Kn* > 1). Figure 16 shows the distribution of the radial component of the convective flow velocity (Fig. 16a) and the corresponding pressure distribution (Fig. 16b) in only part of the cell of Fig. 1b. The flow rate was 140 sccm and plasma effects were not included in this simulation (neutral flow only). The inflow port was modeled as a point source with choked flow at the inlet. One observes that the average flow velocity can exceed 250 m s^−1^ under these conditions. The “plume” of the inflow jet is clearly seen. Also, the flow is relatively very slow in the central region of the Cell. There are substantial pressure gradients in the system. A region of ~ 5×10^−3^ m radius around the inlet port has pressures exceeding 1.33 Pa. The main body of the cell is at around 0.4 Pa, and the exhaust is at less than 0.13 Pa. Detailed DSMC simulations of plasma flow [31] have shown that substantial total density gradients can exist in the reactor due to gas heating (by charge exchange and the Frank-Condon effect) and ion pumping. Ion pumping refers to the phenomenon in which neutrals are ionized in the bulk of the plasma and the resulting ions are driven to the reactor walls by the space charge fields where they recombine turning back into neutrals. Thus the walls are populated with more neutrals compared to the bulk plasma. Plasma-DSMC calculations of the GEC-ICP Cell are currently in progress.

## 7. Summary and Outlook

During the past few years, multidimensional self-consistent plasma simulations which account for the coupled effects of charged and neutral species transport and chemistry have been developed. These simulations vary in their degree of detail from kinetic to fluid to hybrid simulations. Also, different degrees of approximation are used within the same group (e.g., fluid) of simulations. Detailed comparisons with experimental data are necessary to decide which degree of approximation is adequate for accurate determination of some important quantities such as the species density profiles, and the radical and ion flux and energy uniformity along the electrodes. For example, how low in pressure can the fluid approximation be used, and what is the range of pressure and frequency for which the drift-diffusion approximation is correct? Detailed comparisons with data is now actively pursued by many research groups.

For complex chemical systems the accuracy of the simulation may be limited by the lack of knowledge of cross sections for electron-impact reactions and plasma chemistry. In etch or deposition plasmas, knowledge of surface chemistry is viewed as an even more important limitation. This becomes more acute as the operating pressure decreases. Experiments in well characterized systems such as the GEC reference cell, in combination with plasma simulation, will continue to enhance our fundamental knowledge about the plasma dynamics. Many more comparisons with experimental data are needed to “tune” the models and provide simulation tools with predictive capabilities. Considering that 2-D self-consistent rf plasma simulations are only a few years old, such activity is expected to be vigorous in the near future.

The near future will also witness further use of the GEC-ICP cell as high density plasma operation becomes even more important industrially. Also, more studies will be conducted with reactive gas chemistries for wafer etching. The development of diagnostics (especially non-intrusive optical diagnostics) and sensors for real time process control will continue. Although at an early stage of development multidimensional plasma simulations have shown great promise in reproducing many of the dominant features observed experimentally.

## Figures and Tables

**Fig. 1(a):**
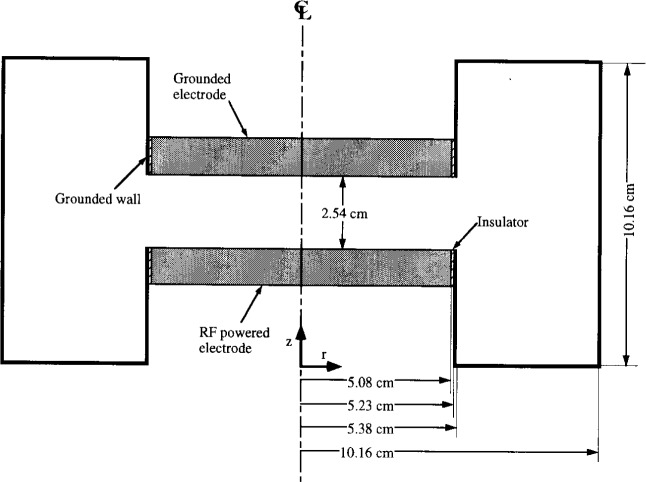
Schematic diagram of the capacitively-coupled GEC Reference Cell.

**Fig. 1b:**
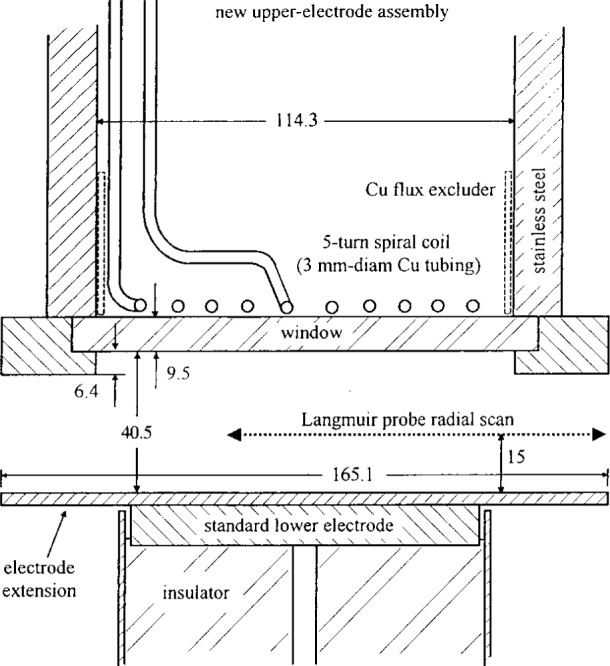
Schematic diagram of the inductively-coupled GEC Reference Cell (Ref. [68]).

**Table 1:** Important collision processes in the agron discharge

No.	Process	Reaction	*H_j_* (eV)	Rate coefficient^a^
1	Ground state excitation	Ar + e →Ar* + e	11.56	*k*_ex_
2	Ground state ionization	Ar + e → Ar^+^ + 2e	15.7	*k*_i_
3	Step-Wise ionization	Ar* + e → Ar^+^ + 2e	4.14	*k*_si_
4	Superelastic collisions	Ar* + e → Ar + e	−11.56	*k*_sc_
5	Quenching to resonant	Ar* + e → Ar^r^ + e		*k*_r_ = 2 × 10^−7^
6	Metastable pooling	Ar* + Ar* → Ar^+^ + Ar + e		*k*_mp_ = 6.2 × 10^−10^
7	Two-Body quenching	Ar* + Ar → 2 Ar		*k*_2q_ = 3 × 10^−15^
8	Three-Body quenching	Ar* + 2 Ar → Ar_2_ + Ar		*k*_3q_= 1.1 × 10^−31^

a Rate coefficients for processes 1–4 are given as a function of electron energy in Fig. 2 of Ref. [19]. Units are cm^3^/s except for *k*_3q_ which is in cm^6^/s.
